# Analgesia Effect of Verum and Sham Acupuncture Treatments in Primary Dysmenorrhea: A MRI Pilot Study

**DOI:** 10.3390/jpm11121244

**Published:** 2021-11-23

**Authors:** Shin-Lei Peng, Hui-Chieh Yang, Yu-Chen Lee, Chun-Ming Chen, Ying-Yu Chen, Cheng-Hao Tu

**Affiliations:** 1 Department of Biomedical Imaging and Radiological Science, China Medical University, Taichung 404332, Taiwan; u104020408@cmu.edu.tw; 2 Department of Acupuncture, China Medical University Hospital, Taichung 404333, Taiwan; d5167@mail.cmuh.org.tw; 3 Department of Medical Imaging, China Medical University Hospital, Taichung 404333, Taiwan; jinmingc@yahoo.com.hk; 4 Department of Chinese Medicine Gynecology, China Medical University Hospital, Taichung 404333, Taiwan; g9512529@oz.nthu.edu.tw; 5 Graduate Institute of Acupuncture Science, China Medical University, Taichung 404332, Taiwan

**Keywords:** cerebral blood flow (CBF), arterial spin labeling (ASL), pain, Sanyinjiao, Streiberger

## Abstract

Acupuncture is an alternative treatment for primary dysmenorrhea (PDM). However, mechanisms by which acupuncture exerts its analgesic properties are still unclear. This study aimed to explore the cerebral blood flow (CBF) response to verum and sham acupuncture treatments, and further investigate whether pre-treatment CBF is capable of assessing symptom changes after interventions. A total of 11 PDM patients in the verum group and 12 patients in the sham group participated in this study. Pain rating index (PRI), CBF, and gonadal hormone levels were acquired before and after 8-week treatments. Both verum and sham acupuncture treatments exert its analgesic effect on PDM after intervention as PRI reduced (*p* < 0.05). Blood gonadal levels were not significantly different after acupuncture in both groups (all *p* > 0.05). In the verum group, intervention-related decreases in CBF were observed in the right dorsal anterior cingulate cortex. In the sham group, regions identified as showing reductions in CBF after acupuncture included the left ventromedial prefrontal cortex, left caudate, and left insula. Patients with higher baseline CBF in the left precuneus and right hippocampus were accompanied with worse treatment response to acupuncture intervention. Mechanisms of verum and sham acupuncture treatments are dissimilar as manifested by different brain responses.

## 1. Introduction

Primary dysmenorrhea (PDM), the most common gynecological disease, is the occurrence of painful menstrual cramping of the uterus, affecting as many as 85% of women. In addition, PDM also increases the risk of fibromyalgia [1]. Standard treatments for PDM including nonsteroidal anti-inflammatory drugs (NSAIDs) and hormonal contraceptive methods are used to exert hypoalgesic action [2,3]. However, side effects such as gastrointestinal complaint and mild neurological symptoms may deter some females from these medications [4]. Acupuncture is a traditional Chinese medicine procedure that has been considered as an alternative treatment for PDM in East Asia for the past 20 years [5]. The National Institute of Health has also recommended acupuncture as an effective treatment for pain alleviation in PDM [6]. Beneficial effects of acupuncture include increasing participation in daily activities and reducing the amount of ingested pain medication [7]. Moreover, one meta-analysis also suggested that acupuncture-related hypoalgesic effects on PDM were more effective compared with NSAIDs [8].

Acupuncture has garnered increased popularity in pain management [9,10]; however, the mechanisms by which acupuncture exerts its analgesic properties are still unclear. Recent research surmised that acupuncture-related hypoalgesic effects may be associated with functional and structural resilience changes in the brain by using neuroimaging techniques such as resting-state functional magnetic resonance imaging (fMRI) [9,11] and voxel-based morphometry [12]. However, to date, information related to the antihyperalgesic effect of acupuncture in terms of cerebral hemodynamics is limited, especially in the study of PDM.

Cerebral blood flow (CBF), which the amount of blood supplied to the brain, provides a direct measurement for quantitatively assessing brain physiology. CBF measurement is advantageous because of its tight regulation to meet the brain’s metabolic demands, which is known as neurovascular coupling. As it has no radiation burden and is capable of recording regional information, CBF obtained by arterial spin labeling (ASL) magnetic resonance imaging (MRI) is considered a valuable strategy for assessing the impact of PDM on brain perfusion. Studies conducted by Zhang et al. using the ASL technique have shown that PDM is associated with CBF abnormalities [13,14]. Therefore, information obtained by analyzing CBF from ASL is beneficial for understanding the underlying mechanisms of PDM. Despite its potential, no study has investigated the possible acupuncture mechanisms for pain alleviation in PDM through ASL.

Previous studies suggested that the treatment effect of acupuncture is also associated with non-specific factors such as expectancy and degrees of efficacy, depending on the participant’s perceptions and expectations [15,16]. Therefore, comparison of sham acupuncture methods is a critical step toward a better understanding of the efficacy and therapeutic mechanisms of acupuncture [9,11,17]. Moreover, as pain is a subjective and personal experience, responses to acupuncture therapies may depend on an individual’s baseline characteristics. Early studies elucidated the functional connectivity circuits at baseline that could be used to evaluate acupuncture treatment response for patients with PDM [9] and chronic low back pain [11]. In light of these studies, a pertinent question arises as to whether analgesic responses in patients with PDM could be explained by the baseline cerebral hemodynamic differences among individuals. The baseline neuroimaging biomarker can shed more light on the PDM mechanism and, to some extent, determine an optimal therapeutic strategy.

Given that the brain perfusion response to acupuncture treatment is missing in the existing literature, the central goal of this study was to explore the CBF response to both verum and sham acupuncture treatments by using the ASL technique in a longitudinal setting. In addition, whether treatment outcomes could be affected by an individual’s baseline characteristics was further investigated. The findings of this study provide a supplementary understanding of the effects of acupuncture on analgesic actions in PDM.

## 2. Materials and Methods

### 2.1. Participants

In this study, 25 right-handed female patients with PDM were recruited through advertisements. The protocol was approved by the local institutional review board, and all participants provided written consent after they were clearly informed of the study protocol. Inclusion criteria for patients with PDM were as follows: (1) age in the range 20–30 years old; (2) regular menstrual cycle of 27–32 days; and (3) average menstrual pain level (including cramping, swelling, tingling, etc.) in the last 6 months rated higher than 4 points on a 10-point visual analog scale (0 = not at all and 10 = the worst pain sensation). The exclusion criteria were as follows: (1) organic pelvic or reproductive system disease(s) found in gynecological ultrasonography; (2) a history of psychiatric or neurologic disorders; (3) current pregnancy or plans for pregnancy; (4) use of oral contraceptives, Chinese herbal medicine, or acupuncture treatment in the past 6 months; and (5) any contraindication for MRI scanning. All patients were required to abstain from any analgesic drugs for at least 24 h prior to the MRI scans.

### 2.2. Experimental Design

Patients were randomly assigned to either verum or sham acupuncture group using a computer-generated list of random numbers. All patients were blinded to the treatment groups, and only the acupuncturist and staffs knew the treatment strategies. To verify the effectiveness of the single blinding procedure, patients were asked whether they had been treated with verum or sham acupuncture after they completed the intervention. All patients believed that they were treated with verum acupuncture.

The ASL was performed before and after acupuncture intervention. MRI scans were performed during a fixed phase of the follicular phase (Days 5–12 of the menstrual cycle) to preclude the influence of the menstrual phase on CBF measurements. The latest menstrual pain experience was assessed using the Chinese version of the McGill Pain Questionnaire (MPQ) [18] following each MRI scan. The score obtained by summing the intensity values for each of the four categories (sensory, affective, evaluative, and miscellaneous) was defined as the pain rating index (PRI). Venous blood samples were taken to assess the concentrations of estrogen, progesterone, and testosterone within 2 days before or after MRI scans using the chemiluminescent immunoassay sandwich method.

### 2.3. Acupuncture Treatment

All acupuncture treatments were performed by a licensed acupuncturist with more than 10 years of experience in Chinese medicinal acupuncture. Each patient received a total of 16 acupuncture sessions within 8 weeks (twice a week), and each acupuncture session lasted approximately 20 min. Acupoints of bilateral Sanyinjiao (SP6), located medially four fingers wide above the ankle, were selected. According to the principles of traditional Chinese medicine, SP6 is the junction point of the liver, spleen, and kidney meridians. By strengthening the spleen and nourishing the liver and kidney, it is believed to be useful for dysmenorrhea relief [19].

The skin of the acupoints was first cleaned with an alcohol swab, and an O-ring was placed on the acupoints and covered with white surgical tape to conceal the penetration depth of the needles. The major difference between verum and sham groups was the “skin penetration.” The verum acupuncture uses the standard acupuncture needles that penetrate the skin of SP6 to a depth of approximately 25 mm, whereas the sham acupuncture was conducted using the Streiberger needles [20] without skin penetration. In both the verum and sham groups, needles were not manipulated during the retention time.

### 2.4. MRI Acquisition

All experiments were performed on a 3T MR system (Signa HDxt, GE, Wisconsin, USA) using a 16-channel head coil. Foam padding was used to stabilize the head to minimize motion. The MRI protocol consisted of a T1-weighted (T1W) fast spoiled gradient echo (FSPGR) and a pseudo-continuous ASL (pCASL) sequence. The scan parameters of the FSPGR sequence were as follows: repetition time (TR)/echo time (TE)/flip angle (FA) = 8.02 ms/2.99 ms/12°, inversion time (TI) = 450 ms, spatial resolution = 1 × 1 × 1 mm^3^, and number of slices = 170. Scan parameters of the 2D pCASL sequence were as follows: TR/TE/FA = 4600 ms/9.8 ms/90°, spatial resolution = 2 × 2 × 4 mm^3^, number of slices = 36, post labeling delay (PLD) = 1.8 s, labeling duration (LD) = 1.5 s, single-shot echo planar imaging, and 30 pairs of label and control images.

### 2.5. MRI Data Processing

The first step of ASL data analysis was to realign control and label images for motion correction using Statistical Parametric Mapping (SPM) software (https://www.fil.ion.ucl.ac.uk/spm/) running in MATLAB (Mathworks, Natick, MA, USA). Label and control pCASL images were pair-wise subtracted and averaged to obtain perfusion weighted images. The quantification of CBF from the perfusion-weighted images was calculated using a model described previously [21]. All the CBF maps were co-registered to the standard template in Montreal Neurological Institute space. The resulting images were smoothed using an 8 mm full-width-at-half-maximum isotropic Gaussian kernel.

### 2.6. Statistical Analysis

To ensure no assumption was made about the probability distribution of the dataset, the non-parametric Mann–Whitney U test was performed on the demographic data, total PRI from MPQ, and blood hormone levels to compare the clinical features acquired from the two groups. The non-parametric Wilcoxon rank test was used to determine whether alterations in the aforementioned parameters were significantly different after treatment in each group. P less than 0.05 was considered significantly.

A two-sample *t*-test from SPM was used to test the region-specific differences in CBF between the groups, with age and gynecologic age as covariates. Here, CBF before and after treatment was tested. A paired *t*-test was performed for each group to assess voxewise differences in CBF before and after treatment using SPM. Predicted activations were considered significant at *p* < 0.005 (uncorrected) and clusters of more than 100 voxels. Furthermore, clusters of contiguous voxels classified as significant were extracted as regions of interest (ROIs). The averaged CBF within the ROI was calculated for each subject, and the differences in CBF before and after treatment were compared using the non-parametric Wilcoxon rank test. Less than 0.05 was considered significant. In addition, two treatment groups were combined as a pooled group to investigate whether the acupuncture treatment response could be affected by an individual’s baseline CBF. A general linear model was used with changes in pain severity (pre-treatment-post-treatment) as a dependent variable, and baseline CBF, treatment method, and age were independent variables.

## 3. Results

### 3.1. Demographic and Clinical Information

Among the twenty-five participants, one patient dropped out during the intervention session and another was excluded owing to image artifacts, leaving eleven patients in the verum group and twelve patients in the sham group. No significant differences were found in gynecologic age, body mass index, length of menstrual cycle, or dysmenorrhea history (all *p* > 0.05). Demographic information of the patients is listed in Table 1. The gonadal hormone levels of estrogen, progesterone, and testosterone are shown in Table 2. The blood gonadal concentrations were not significantly different before and after acupuncture interventions in both groups (all *p* > 0.05), suggesting that acupuncture does not affect hormone levels.

**Figure 1:**
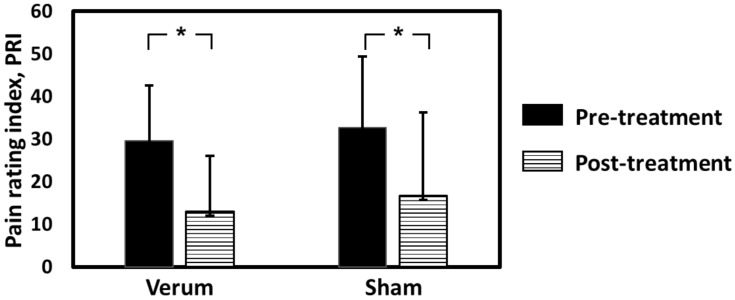
Total pain rating index from McGill Pain Questionnaire before and after acupuncture treatments. * *p* < 0.05.

The results of total PRI from MPQ are displayed in Figure 1. In the verum group, the values of total PRI before and after intervention were 29.5 ± 13 and 19.2 ± 13, respectively. In the sham group, the values of total PRI before and after intervention were 32.6 ± 16.7 and 20.6 ± 19.4, respectively. The values of total PRI significantly decreased in both groups (both *p* < 0.05), suggesting the analgesic effect of both verum and sham acupuncture on PDM after 8 weeks of intervention. The total PRI after intervention was not significantly different between groups (*p* = 0.73), suggesting that the treatment effect was comparable between groups.

### 3.2. CBF Voxelwise Analyses

**Figure 2:**
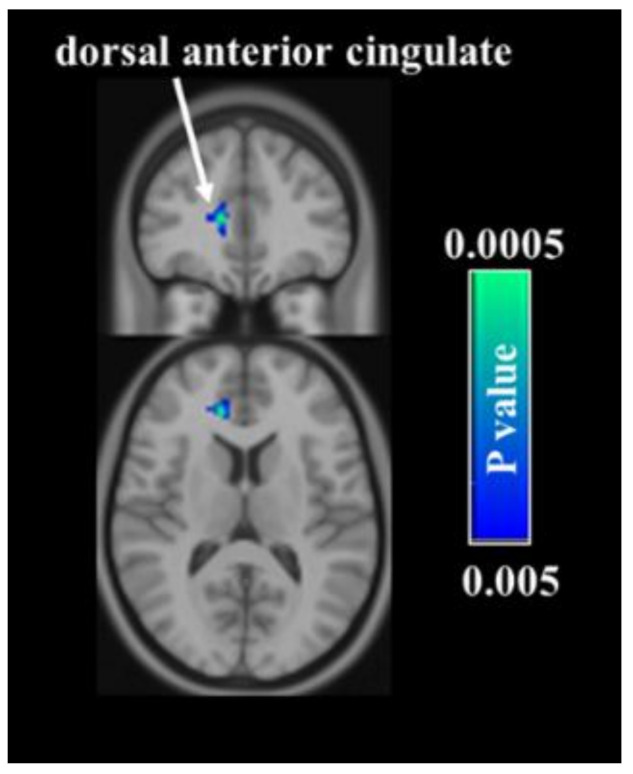
Comparison of cerebral blood flow (CBF) between two time-points in the verum group. Voxelwise analyses demonstrate a decrease in CBF in the right dorsal anterior cingulate cortex after treatment.

**Figure 3:**
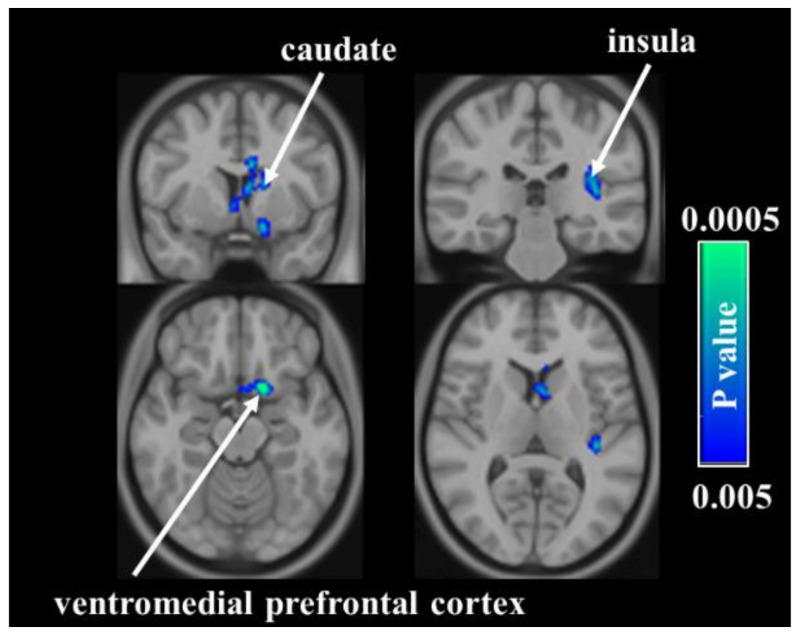
Comparison of cerebral blood flow (CBF) between two time-points in the sham group. Voxelwise analyses demonstrate decreases in CBF in the left ventromedial prefrontal cortex, left caudate, and left insula.

**Figure 4:**
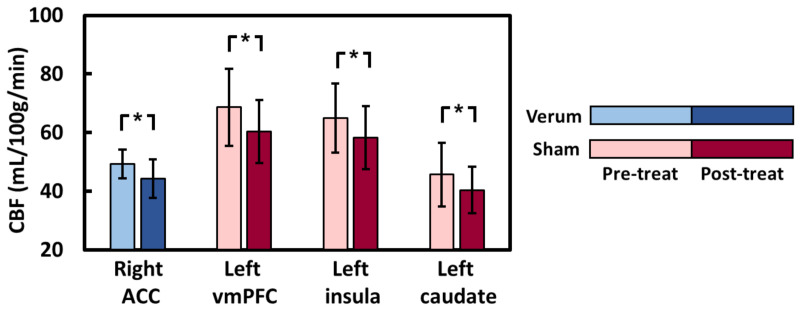
Regions of interest analysis for cerebral blood flow before and after acupuncture treatment. *: *p* < 0.01.

The regional CBF maps measured with ASL MRI were then analyzed to examine which brain regions manifested acupuncture-associated alterations. In the verum group, voxelwise analyses suggested that intervention-related decreases in CBF were most significant in the right dorsal anterior cingulate cortex (dACC) (Figure 2). In the sham group, regions identified with CBF reductions after acupuncture included the left ventromedial prefrontal cortex (vmPFC), left caudate, and left insula (Figure 3). In terms of ROI analysis (Figure 4), CBF in the aforementioned regions decreased significantly after treatment (all *p* < 0.01). The opposite result, i.e., acupuncture-related increase in CBF, was not detected in either group. The CBF between verum and sham groups did not differ significantly before and after treatments.

### 3.3. Regression Analysis between Changes in Total PRI and Baseline CBF

**Figure 5:**
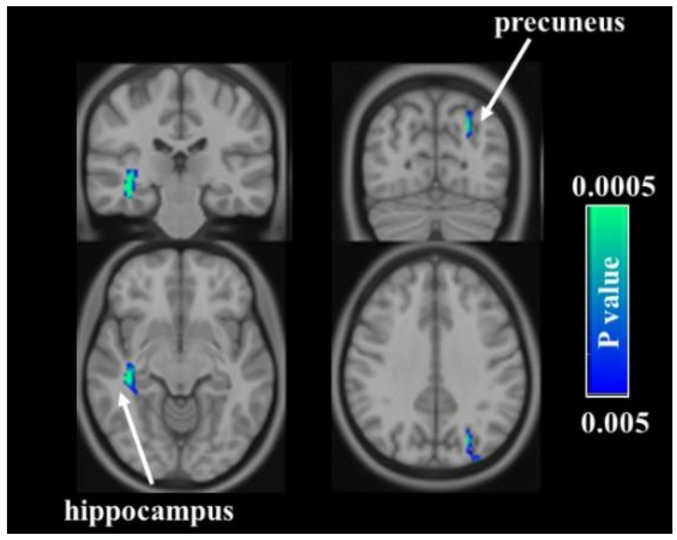
Regression analysis for the relationship between analgesic relevance and baseline cerebral blood flow (CBF). The higher baseline CBF in left precuneus and right hippocampus accompanied with worse treatment response in patients with primary dysmenorrhea.

To investigate the analgesic relevance following acupuncture, we correlated the pre-post changes in total PRI score with the baseline CBF for the pooled group. After controlling for age and treatment group, the left precuneus and right hippocampus showed a significantly negative correlation between changes in clinical pain and baseline CBF (Figure 5), suggesting that higher baseline CBF in these regions was associated with worse treatment response in patients with PDM.

## 4. Discussion

This exploratory study investigated the cerebral hemodynamic response to acupuncture treatment in PDM using the neuroimaging biomarker of CBF. Our results show that acupuncture exerting hypoalgesic action might not be mediated by the hormone levels in patients with PDM. Instead, acupuncture might reverse chronification by decreasing CBF to mediate the perception of nociceptive input. Moreover, the mechanisms of verum and sham acupuncture treatments are dissimilar, as manifested by different brain responses. Both verum and sham acupuncture treatments demonstrated a salutary effect on PDM; however, the changes in pain severity depended on the baseline CBF, with patients having higher resting perfusion in the precuneus and hippocampus but minimal response to acupuncture treatment. The findings in this study provide new information on tracking the impact of the antinociceptive characteristics of acupuncture on PDM using the brain perfusion technique.

The reduced CBF observed in the dACC after verum acupuncture treatment is intriguing and warrants further investigation. Verum acupuncture can stimulate the afferent fibers of peripheral nerves and elicit De-Qi sensation [22], and it is believed to achieve its analgesic effect by activating the antinociceptive system, for instance through increased opioid receptor availability [17,23]. A positive correlation between opioid receptor availability and pain alleviation was demonstrated in a previous study [24]. Moreover, opioid receptors are well known to contribute to the vasoconstriction effect, and direct activation of opioid receptors causes sustained decreases in cerebral blood volume (CBV) [25]. Given that the experience of chronic pain is the result of complex neurological processes [26], chronic pain generally activates dACC [27], which has been implicated in circuits involved decision making, emotional processing, learning, and sensory attention [28,29,30,31]. In this regard, decreased CBF in dACC after verum acupuncture intervention could be attributed to the involvement of vasoactive neurotransmitters in this brain region [17] to exert multiple influences on pain relief. This tuning-down effect from verum acupuncture could lead to reappraising the nociceptive memory and reducing aversive prediction, resulting in shifting attention away from incoming pain.

A sham acupuncture strategy is an essential step toward understanding the efficacy and potential therapeutic mechanisms of acupuncture. The procedure of sham acupuncture used in this study provides a sensation on acupoints but does not penetrate the skin, and is indistinguishable from typical verum acupuncture [20,32]. In terms of pain alleviation, sham acupuncture was established to be as effective as verum acupuncture in previous studies [33,34]. The same conclusion was drawn in this study. Given that the peripheral nerves were not stimulated, the significant reductions in CBF found in other pain-associated regions, including the caudate, insula, and vmPFC in response to sham acupuncture may not be generally attributed to a mechanism similar to that of verum acupuncture [17]. The sensory experience of skin stimulation combined with psychological expectation in the belief of acupuncture was suggested to have an important role in shaping placebo analgesia in acupuncture [12,35]. Dopaminergic reward circuitry engages in shaping expectations [36], and recent studies linked increasing dopamine D2/D3 receptor availability in the caudate to placebo hypoalgesia [37]. Moreover, the upregulation of dopamine D2/D3 receptors leads to vasoconstriction [38], thereby causing a notable CBF reduction. In another aspect, the insula is a key region involved in the cognitive modulation of pain and sensory-discriminative components of pain [39]. Neuroimaging studies indicate that reduced pain ratings during placebo analgesia are often coupled with decreased activity in the insula [40,41]. In particular, CBF was shown to tightly correlate with neuronal activity [42]. Thus, results of decreased CBF in the insula in this study may suggest that sham acupuncture exerts its analgesia by inhibiting the neuronal activity in the insula to modulate pain experience. The vmPFC is another brain region linked to cognitive pain modulation, and is also a brain region engaged in expectation-induced analgesia [43]. Increased activation in vmPFC was observed during pain perception [44,45], but interestingly, placebo-induced analgesia is linked to enhanced activity in vmPFC [46], in contrast with the finding of reduced CBF in vmPFC after sham acupuncture intervention in this study. A clear-cut explanation was not obtained for this observation, but possible reasons for this discrepancy may be associated with differences in pain stimulus and treatment strategies.

Verum and sham acupuncture exert hypoalgesic action through different mechanisms, as manifested by different patterns of brain responses; however, our findings also provide some information on shared characteristics evoked by each type of treatments. First, both strategies achieve analgesic effects by decreasing CBF to some extent, thus inhibiting neural activity. Accumulated evidence indicates that patients with PDM present an aberrant increase in neuronal activity. For instance, using resting-state fMRI, Liu et al. found increased spontaneous neural activity in the ACC in patients with PDM versus healthy ones [47]. A study based on positron emission tomography study by Tu et al. showed that patients with PDM increased their cerebral metabolism in the prefrontal regions [48]. Given that acupuncture action involves the interaction between neurotransmitters and modulators, deactivation indicates a redistribution of attention resource [49] and therefore balances pain-related central networks. Second, gonadal hormone levels contribute to the pathogenesis of dysmenorrhea [50], and oral progesterone [2] or estradiol valerate [3] bring immediate relief from symptoms associated with PDM; however, gonadal hormone levels did not change after acupuncture treatments [19] in either group. Based on the results of this study, we tentatively suggest that the analgesic effect of acupuncture might not be mediated by alterations in hormone levels. Instead, direct engagement of brain circuits plays a crucial role in pain chronification.

Although the reduction in pain severity was comparable between verum and sham treatments in this study, existing studies examining the effects of sham acupuncture on PDM have typically produced mixed results [9,19,51]. Three different types of sham acupuncture treatments have been proposed in the literature: (1) using the Streitberger placebo acupuncture needle at the acupoints without skin penetration [11]; (2) using a real acupuncture needle at the acupoints considered specific for another disease [19,51]; and (3) using a real acupuncture needle at the non-acupoints [9,51]. In this study, sham acupuncture treatment was achieved using the Streitberger placebo acupuncture needle at the acupoints without skin penetration. This strategy evoked a similar reduction in pain when compared with verum acupuncture, and the treatment efficiency of Streitberger placebo acupuncture has also been established in other studies [11]. This raises a fundamental question regarding whether acupuncture needles must be inserted at specific acupoints to have their greatest effects [51]. Moreover, as the Streitberger needle causes a pricking sensation, it might not be completely inactive [52]. Collectively, debates on sham acupuncture treatments in PDM are ongoing and welcomed. As we are only at an early stage in our attempt to understand the mechanisms behind the acupuncture treatments by using neuroimaging techniques, comparing with other sham acupuncture treatments such as different acupoints would help to better understand these questions.

The experience of pain-related sensation is derived from the complicated evolution of afferent information arising from peripheral sensory receptors to pain-related brain regions for signal processing. Consequently, treatment outcomes have been proposed to be affected by an individual’s baseline characteristics. Indeed, our results demonstrated that a higher baseline CBF in the precuneus and hippocampus was associated with a worse treatment response in patients with PDM. Both the precuneus and hippocampus are the key nodes of the default mode network (DMN), and recent studies suggested that hypertrophic alterations of the precuneus and hippocampus may be intricately involved in the pathophysiology of PDM. Relative to normal participants, patients with PDM showed an aberrant increase in resting CBF and gray matter volume in the precuneus [13] and hippocampus [53], respectively. Moreover, patients with PDM displayed enhanced precuneus [47] and hippocampus [54] spontaneous neural activity as measured by resting-state fMRI. The DMN plays a significant role in memory consolidation and is the primary network affected by chronic pain [55]. Chronic pain has been conceptualized as a type of learning behavior [56]. If long-term accumulation of nociceptive memory of PDM has disrupted the dynamics of brain plasticity in precuneus and hippocampus due to the hypertrophic alterations, no nociceptive memory updates, thereby reducing the pain relief response to treatment.

The results of this study must be interpreted in light of some limitations. First, CBF did not differ significantly between groups after treatments, and this could be partially attributed to the relatively small sample size in this study. Future studies with a larger sample size could be an essential step toward a better understanding of brain alterations after acupuncture treatment in patients with PDM. Second, all participants underwent MRI scans after an 8-week treatment. The long-term effect of acupuncture was not explored. Third, the present study only explored the “trait pain” at the follicular phase while symptoms were absent. Given that the PDM is a cyclic chronic pain, scanning participants during the “state pain” at the menstrual period could shed more light on the analgesic effect of acupuncture. The fourth limitation was that no control group was evaluated in this study. As acupuncture treatment requires a significant amount of time and there is no motivation for the control group to receive acupuncture treatments if they have no symptoms, the recruitment of control subjects was a challenge in this study and in other neuroimaging studies [9,10,11,12,17].

## 5. Conclusions

In conclusion, the data presented in this study showed that both verum and sham acupuncture can exert analgesic effects by decreasing CBF to mediate the perception of nociceptive input in patients with PDM, but mechanisms should be different as manifested by different pattern of brain responses. In addition, a higher baseline CBF in the precuneus and hippocampus were accompanied by a worse acupuncture treatment response. Collectively, findings of this study support the idea that CBF from ASL technique can be considered as a useful neuroimaging biomarker for investigating different brain mechanisms underlying verum and sham acupuncture in patients with PDM.

## Figures and Tables

**Table 1:** The demographic information in both groups. All data are presented as men ± SD.

	Verum (*N* = 11)	Sham (*N* = 12)	*p*-Value
Age (year)	22.55 ± 2.34	25.75 ± 4.52	0.09
Gynecologic age (year)	11.36 ± 2.42	13.25 ± 5.26	0.32
Body mass index ((kg/m^2^)	21.94 ± 4.68	20.46 ± 1.65	0.60
Length of menstrual cycle (day)	30.36 ± 2.25	29.67 ± 2.27	0.42
Dysmenorrhea history (year)	6.73 ± 3.55	9.92 ± 5.28	0.09

**Table 2:** Gonadal hormone levels before and after acupuncture treatments. All data are presented as mean ± standard deviation.

	Verum			Sham		
	**Pre**	**Post**	***p* Value**	**Pre**	**Post**	***p*-Value**
Estradiol (pg/mL)	139.82 ± 110.08	141.27 ± 82.92	0.83	138.17 ± 88.89	132.75 ± 109.10	0.91
Progesterone (ng/L)	1.59 ± 3.24	4.09 ± 6.85	0.41	1.08 ± 1.30	2.38 ± 4.51	0.56
Testosterone (ng/mL)	0.61 ± 0.22	0.60 ± 0.22	0.95	0.57 ± 0.11	0.50 ± 0.14	0.06

## Data Availability

The data can be freely given upon request.
